# Supplementary figures and images for: Prolonged Sleep Latency and Reduced REM Latency Are Associated with Depressive Symptoms in a Japanese Working Population
Source: Int J Environ Res Public Health. 2022 Feb 13;19(4):2112. doi: 10.3390/ijerph19042112 (PMC8872621; doi:10.3390/ijerph19042112)

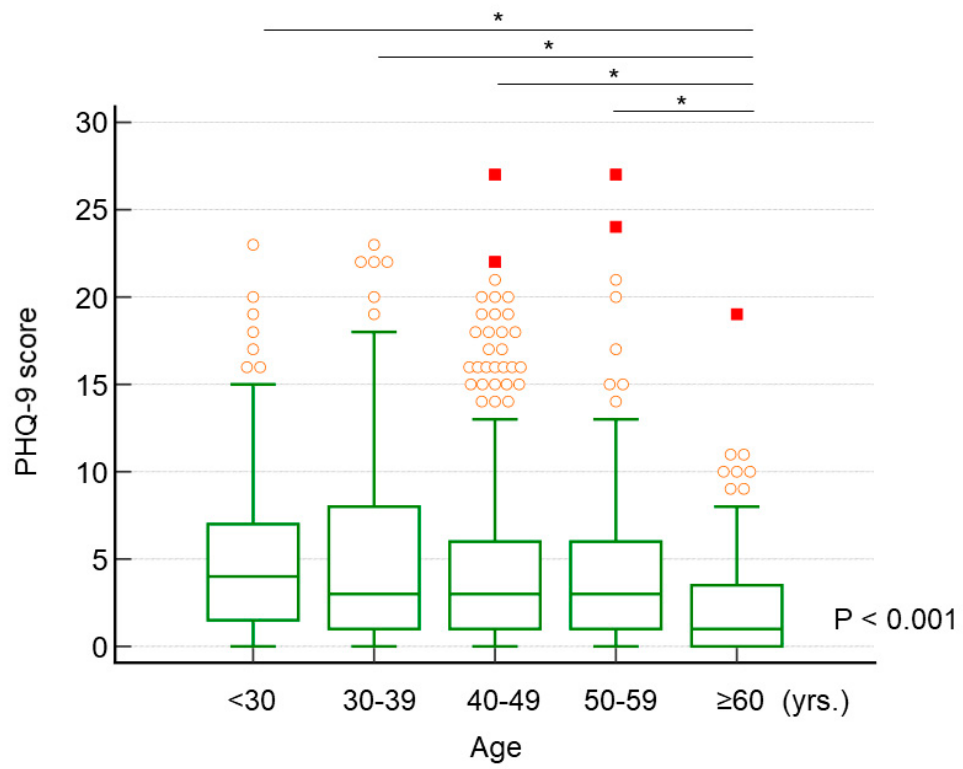

**Figure S1.** PHQ-9 scores vs. age.

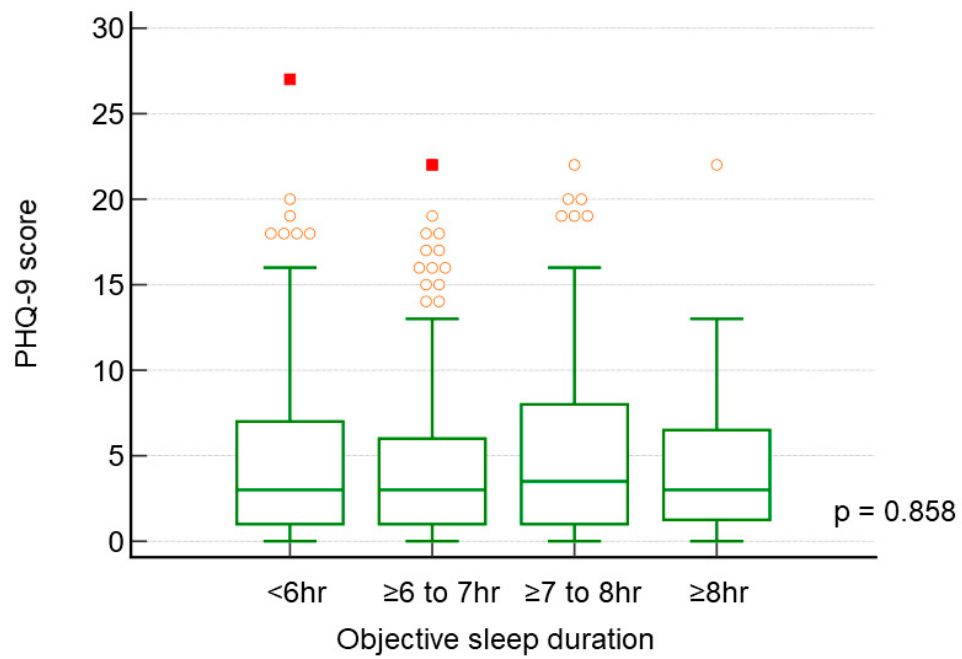

**Figure S2.** PHQ-9 scores vs. SPT.

Supplement: Supplementary file 1 [file ijerph-19-02112-s001.zip › ijerph-1569546-supplementary.pdf]
